# Network target for screening synergistic drug combinations with application to traditional Chinese medicine

**DOI:** 10.1186/1752-0509-5-S1-S10

**Published:** 2011-06-20

**Authors:** Shao Li, Bo Zhang, Ningbo Zhang

**Affiliations:** 1 MOE Key Laboratory of Bioinformatics and Bioinformatics Division, TNLIST / Department of Automation, Tsinghua University, Beijing 100084, China

## Abstract

**Background:**

Multicomponent therapeutics offer bright prospects for the control of complex diseases in a synergistic manner. However, finding ways to screen the synergistic combinations from numerous pharmacological agents is still an ongoing challenge.

**Results:**

In this work, we proposed for the first time a “network target”-based paradigm instead of the traditional "single target"-based paradigm for virtual screening and established an algorithm termed NIMS (Network target-based Identification of Multicomponent Synergy) to prioritize synergistic agent combinations in a high throughput way. NIMS treats a disease-specific biological network as a therapeutic target and assumes that the relationship among agents can be transferred to network interactions among the molecular level entities (targets or responsive gene products) of agents. Then, two parameters in NIMS, Topology Score and Agent Score, are created to evaluate the synergistic relationship between each given agent combinations. Taking the empirical multicomponent system traditional Chinese medicine (TCM) as an illustrative case, we applied NIMS to prioritize synergistic agent pairs from 63 agents on a pathological process instanced by angiogenesis. The NIMS outputs can not only recover five known synergistic agent pairs, but also obtain experimental verification for synergistic candidates combined with, for example, a herbal ingredient Sinomenine, which outperforms the meet/min method. The robustness of NIMS was also showed regarding the background networks, agent genes and topological parameters, respectively. Finally, we characterized the potential mechanisms of multicomponent synergy from a network target perspective.

**Conclusions:**

NIMS is a first-step computational approach towards identification of synergistic drug combinations at the molecular level. The network target-based approaches may adjust current virtual screen mode and provide a systematic paradigm for facilitating the development of multicomponent therapeutics as well as the modernization of TCM.

## Background

Multicomponent therapeutics, in which two or more agents interact with multiple targets simultaneously, is considered as a rational and efficient form of therapy designed to control complex diseases [1,2]. Here “agent” refers to medicinal entities, chemical substances, herbs and the like with pharmacological or biological activities. One of the fundamental advantages of multicomponent therapeutics is the production of “synergy”, that is, the combinational effect to be greater than the sum of the individual effects, making multicomponent therapeutics a systematic approach, rather than the reductionism of an additive effect. Understanding multicomponent synergy is critical for developing a novel strategy to conquer complex diseases. It is believed that combinations of agents can effectively reduce side effects and improve adaptive resistance, thereby increasing the likelihood of conquering complex diseases, such as cancer, in a synergistic manner [3].

Evaluation of multicomponent synergy is usually implemented experimentally in a case-by-case approach [4] and evaluated using the reference models of additivism to recognize synergy such as the Bliss independence model [5], the Loewe additivism model [6] and the Combination Index theorem [7]. However, large number of possible agent combinations will be formed even in the case of a small collection of therapeutic agents. Therefore, although some experimental methods have been launched to screen favourable drug combinations by disease-relevant phenotypic assays [8], the high-throughput identification of synergistic agent combinations arising from numerous agents remains an unresolved issue [9]. By way of contrast, computational approaches that take advantage of the rapid accumulation of massive data may provide a more promising and desirable method for multicomponent drug studies. Currently, computational efforts for the evaluation of multicomponent therapeutics mainly focus on two directions. The first direction is to identify and optimize multiple target interventions by modelling signaling pathways or specific processes and is usually applied to small scale problems [10,11]. One of limitations of this approach is the fact that crosstalks, feedbacks or interactions among pathways are widely present in complex diseases, suggesting that pathways should be integrated rather than treated separately [12,13]. The second newly developing direction is to measure the efficacy of drugs, especially multi-target drugs, by using network biology approaches [14]. However, the realistic method remains to be established and the association between drug actions and network properties is not precisely known. Thus, finding ways to evaluate multicomponent therapeutics and sort order for synergistic agent combinations is still a considerable challenge. Novel computational approaches are urgently required for feasible and efficient identification of multicomponent synergy.

Recently, computational systems biology approaches as well as our previous studies have been enhancing our understanding of various aspects of complex diseases, including the identification of disease-related genes or functional modules, and the recognition of redundant, adaptable and system mechanisms in diseases [15-17]. Now, we are standing at the portal of a new era to bridge molecular states to physiological states as well as various disease states through the biological networks that sense genetic and environmental perturbations [18]. To keep in line with new developments, researchers have also started to change their way of thinking in terms of drug-treated complex biological systems, and studies such as network pharmacology [19] have been springing up. Against this background, we propose a novel concept, “network target”, with the attempt to update current single target-based or multiple target-based drug studies. We roughly defined the “network target” as a therapeutic target that is derived from systematic interventions of the biological network (including the network state and its pivotal elements) underlying a disease or pathological process. The concept of network target considers simultaneously the disease mechanisms and drug actions on a network basis, and a network target for a certain disease may correspond to a variety of single-component or multicomponent therapeutics.

On the other hand, while the scientific community has high expectations for the coming network pharmacology [19], this new field should be composed of two main approaches due to our poor understanding of cell behaviours and drug-protein interactions: 1) Bottom-up: Addition of well-known molecular drugs and observation of synergistic effects; 2) Top-down: Reduction of more general formulae to its minimal elements that keep its beneficial properties. In this regard, an empirical system of multicomponent therapeutics, traditional Chinese medicine (TCM), may have the potential of addressing a relationship between multicomponents and drug synergistic effects. Having been evolved over 3,000 years, TCM is characterized by the use of Herbal Formulae (*Fu-Fang*) that are usually grouped by two or more medicinal herbs and capable of systematically controlling various diseases such as angiogenic disorders [20] via potentially synergistic herb interactions [21,22]. For instance, the Realgar-*Indigo naturalis* Formula has an effect on promyelocytic leukemia via the action mechanism of synergy among its components [23]. Thus, the multicomponent synergy in Chinese herbs is of great significance for understanding TCM and for new drug discovery. Although this is still an open question, it is believed that the rich body of TCM experience in combined use of herbs may provide an excellent model for studying synergistic effects among different components [24], and the systems biology approaches could shed light on the mystery of TCM [22,25].

In this work, we report a novel method, called NIMS (Network target-based Identification of Multicomponent Synergy), to address the network target-based virtual screen and assess the synergistic strength of multicomponent therapeutics. NIMS measures synergistic agent combinations by creating and integrating two parameters, namely Topology Score and Agent Score. Next, NIMS was applied to prioritize synergistic combinations from 63 agents including 61 herbs or herb compounds as well as five agent pairs with known synergistic effects containing 2 additional chemicals 5-fluorouracil and Rapamycin. One of NIMS outputs was then subjected to experimental verification. We hope the network target-based approaches will improve our understanding of multicomponent therapeutics in terms of complex biological systems.

## Results

### Pipeline of NIMS

The rationale of the network target concept and NIMS is to transfer the relationship among agents to the interactions among the targets or responsive gene products of agents in the context of a biological network specific for a disease or pathological process. This hypothesis may be reasonable in many situations especially when synergy occurs only if the effects of individual agents are mediated through independent action mechanisms. In NIMS, a set of genes or gene products affected by an agent are termed *agent genes*, and the disease-specific biological network serves as the background network to perform NIMS. Then, two elements in NIMS, Topology Score (*TS*) and Agent Score (*AS*), are proposed to evaluate agent interactions.

**Figure 1:**
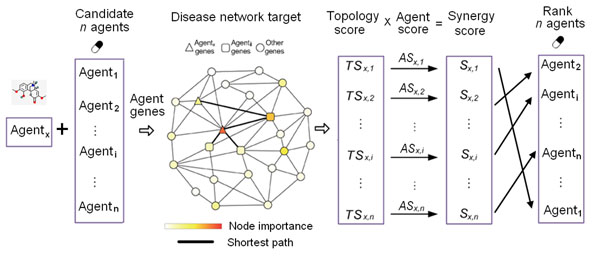
**Pipeline of NIMS: ranking the synergistic effect of *n* agents paired with a given agent.** For a given agent (*Agent_x_*) and *n* candidate agents (*Agent_1_*, …, *Agent_n_*), all *agent genes* are collected and mapped to a disease network target. For each agent (*Agent_1_*, …, *Agent_n_*) combined with *Agent_x_*, *TS* (Topology Score) is obtained by calculating the node importance of both sets of *agent genes* and the shortest path between them. *TS* is subsequently weighed by the *AS* (Agent Score) of each agent pair to ultimately produce *S* (Synergy score), which is used to rank the synergy strength for the *n* candidate agents matched with the given *Agent_x_*.

As shown in Figure 1, *TS* is derived from topological features of the background network related to certain disease conditions and drug actions. From the network target perspective, the achilles’ heel of the biological network underlying a certain disease is more likely to become the attack points of drugs. Thus, we assume that the more important the *agent gene* as a network node is, the stronger effect on the disease the agent will produce. To determine the importance of an *agent gene* as a node in the network, we propose a node importance score, (*IP*(*v*), here *v* denotes a vertex / node), by integrating degree [26], betweenness [27] and closeness [28], three network centrality indexes that have been used to define the network properties of drug targets separately or collectively [29]. Moreover, we suppose that if an agent pair produces synergy, their *agent genes* should be adjacent in the network. Accordingly, for a candidate agent pair *agent_1_* and *agent_2_*, we defined a topology-dependent score, *TS*, to evaluate both the importance score (*IP*(*v*)) of *agent_1_ genes* and *agent_2_ genes* and the network distance between these two gene sets. *TS*_1,2_ is given by:

where *IP*_1_(*i*) for *agent_1_ genes* and *IP*_2_(*j*) for *agent_2_ genes* are calculated by integrating Betweenness, Closeness and a variant of the Eigenvector PageRank [30] through Principal Component Analysis (PCA). The negative exponential function is utilized to weigh the interaction of two agents based on the shortest path length. The min(*d_i_*_,_*_j_*) is the minimum shortest path from *gene_i_* of *agent_1_* to all *agent_2_ genes*, whereas min(*d_j_*_,_*_i_*) is the minimum shortest path from *gene_j_* of *agent_2_* to all *agent_1_ genes*. We only consider the nearest connection between *agent_1_ genes* and *agent_2_ genes* in the network. The two terms in the brackets are dual and represent the synergy strength measurement for a combination of *agent_1_* and *agent_2_*.

As agents with independent action mechanisms but treating similar diseases may be more likely to produce synergistic effect, we also introduced *AS*, a concept transferred from the disease phenotype similarity [31], to quantify the similarity score of two agents and fine-tune the *TS* results. Here, if an *agent gene* falls into the gene set of a phenotype recorded in the OMIM (Online Mendelian Inheritance in Man) database, this phenotype will be identified as an *agent phenotype* for the given agent. The similarity between two *agent phenotypes* quantifies the overlap of their OMIM descriptions and is calculated by a text mining method [31] (**See Methods**). The *AS* for *agent_1_* and *agent_2_* is given by , where *P_i_*_,_*_j_* is the similarity score between *phenotype_i_* of *agent_1_* and *phenotype_j_* of *agent_2_*, and *N* is the total number of phenotype pairs.

Ultimately, NIMS produces the synergy score, *S*_1,2_, for *agent_1_* and *agent_2_* by calculating *S*_1,2_ = *TS*_1,2_ × *AS*_1,2_, which denotes the node importance, network adjacency and action similarity of two gene sets of *agent_1_* and *agent*_2_. A high score means a great probability of synergy. Note that currently NIMS only measures the synergy of combinational agents with independent mechanisms according to the Bliss independent theory [5], so we roughly set the valid range of the NIMS score from 0 to 0.9. When the score is larger than 0.9, the two agents in combination are more likely to act on the same gene sets and in contradiction with the independence assumption. For these agent combinations, we may need more information to distinguish their interaction modes.

### Application and experimental verification of NIMS

We applied NIMS to prioritize synergistic agent pairs from 63 manually collected agents (**See Methods**) and estimated their effects on angiogenesis, a key pathological process in various diseases such as cancer and rheumatoid arthritis [32], with the network constructed by our LMMA approach previously [17]. The NIMS synergy scores for all agent pairs against the angiogenesis network ranged from 0.199270 to 0.012959, with *TS* score from 0.814868 to 0.103790 and *AS* score from 0.262459 to 0.107882, respectively. From the outputs of NIMS, we firstly checked the rank of five agent pairs with known synergy in every 62 pairs for a given agent. As shown in Table 1, the synergy scores of both 5-fluorouracil (5-FU) combined with Vinblastine [33] and 5-FU combined with Rapamycin [34] entered the top three. Three other synergistic pairs, Vinblastine and Camptothecin [35], Genistein and Camptothecin [36], and Genistein and Rapamycin [37], also earned high marks and ranked in the top layer. We then used, respectively, three global background networks including the global protein-protein interaction (PPI) network and two kinds of global pathway networks (Keep Node Content and Merge Node Content, KNC and MNC) (**See Methods**) to calculate the synergy score. Results showed that NIMS is relatively robust to different background networks in these cases (Table 1).

**Table 1:** NIMS ranks against four types of background networks

		Rank among 62 agent pairs #			
		
Given agent	Partner agent	Angiogenesis network (NIMS score)	PPI	KNC	MNC
5-fluorouracil	Vinblastine*	2 (0.18104)	1	2	2
	Rapamycin*	3 (0.13744)	2	3	26

Vinblastine	Camptothecin*	1 (0.19927)	1	1	3

Genistein	Camptothecin*	2 (0.12070)	3	2	2
	Rapamycin*	6 (0.11533)	4	7	4

Sinomenine	Matrine	4 (0.10923)	6	3	11
	Honokiol	8 (0.10142)	5	9	16
	Luteolin	10 (0.10007)	11	17	6
	Quercetin	14 (0.09835)	20	5	3
	Paeoniflorin	29 (0.08215)	26	29	31

*: Agent pairs with known synergistic effects.

#: For each given agent, there are totally 62 candidate agent pairs. PPI, protein-protein interaction network. KNC, Keep Node Content pathway network. MNC, Merge Node Content pathway network.

**Figure 2:**
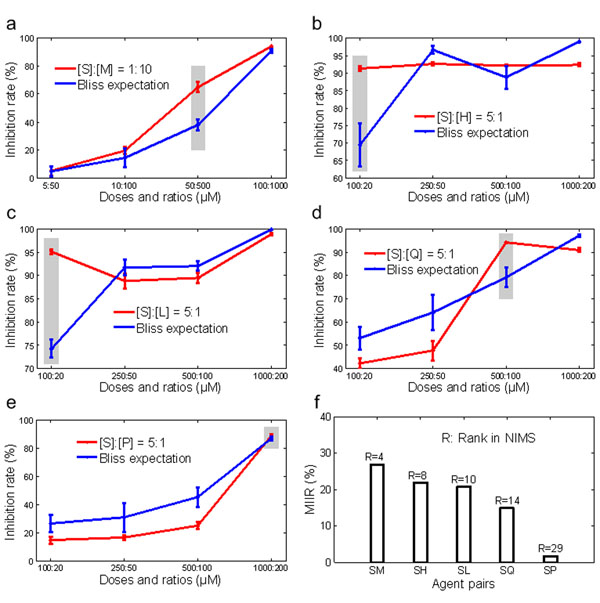
**Anti-angiogenesis synergistic effects of five agent pairs. a-e.** The red line denotes the inhibition rate of Human Umbilical Vein Endothelial Cells (HUVEC) proliferation in a dose-dependent manner. The blue line denotes the additive effects calculated by the Bliss independence model. The gray column denotes the optimal dose and ratio of each pair. **f**. The value of the maximum increased inhibition rate (MIIR) for the synergistic effects produced by five agent pairs corresponds well with the NIMS ranks against the angiogenesis network. The proportion of two agents is determined by following the same ratio of the two agent’s IC50 values.

Next, an *in vitro* assay was conducted to validate NIMS predictions. Sinomenine, an anti-angiogenic alkaloid that extracted from a TCM commonly used herb named *Sinomenium acutum*[20,38], was selected as the seed agent (as *Agent_x_* in Figure 1). Agent combinations were sampled from five intervals of the rank list composed of all 62 agents matched with Sinomenine. Here, we only considered commercially available agents with known chemical structures. This restriction left five Sinomenine partners, namely Luteolin, Quercetin, Honokiol, Matrine and Paeoniflorin. To determine the synergy strength of the agent pairs, low-dose combinations with more than a 70% inhibition rate were regarded as effective [39]. Using the Maximum Increased Inhibition Rate (MIIR) measure for each combination (Figure 2), we found that the highest MIIR 26.83% was reached by Sinomenine combined with Matrine ((S):(M)), whereas the lowest MIIR 1.86% was reached by Sinomenine combined with Paeoniflorin ((S):(P)). This rank order of agent pairs is identical to the order predicted by NIMS when against the angiogenesis network, and such a performance is superior to those against three global networks (Table 1).

### Robustness of NIMS

NIMS integrated three measures, namely Betweenness, Closeness and PageRank to capture the node importance *IP*(*v*) from different aspects. In the undirected angiogenesis network, we found that all three measures are highly correlated and the majority (94.81%) of their variance can be explained by the primary eigenvector. However, these three centrality measures could not replace one another, especially in the directed networks. Thus, we integrated these three centrality measures to address the node importance from different angles. Furthermore, the positive role of *AS* in NIMS was also shown in the agent pair rankings. In the case study, the *AS* scores of Matrine, Honokiol, Luteolin, Quercetin and Paeoniflorin separately combined with Sinomenine were 0.1708, 0.1590, 0.1705, 0.1611 and 0.1414, respectively. These scores reached an approximate rank with that resulted from network topologies alone. The removal of the *AS* scores ranked Luteolin ahead of Quercetin, suggesting that the integration of *AS*, which reflects current knowledge about complex diseases and agent actions, could improve the predictive accuracy of NIMS by weighing *TS*.

**Figure 3:**
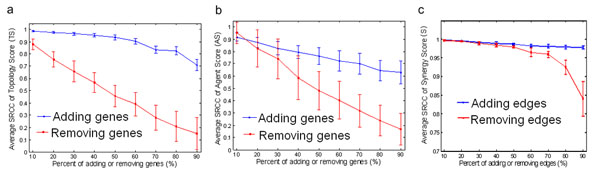
**Permutation tests to assess the robust performance of NIMS.** The permutations are performed by evaluating fluctuations of (**a**) *TS* (Topology Score), (**b**) *AS* (Agent Score), and (**c**) the background network (angiogenesis network) and calculated by the average SRCC (Spearman rank correlation coefficient) between the permutation outputs and the original scores.

The robustness of NIMS was also addressed with respect to both *agent genes* and the background network. By adding or removing *agent genes* randomly, the permutation test results showed that the Spearman Rank Correlation Coefficient (SRCC) was relatively stable when adding genes, but the SRCC decreased dramatically when some key genes were removed (Figure 3a and Figure 3b). The results evidence that the NIMS synergy score may be determined largely by some key *agent genes*, and the rank results will remain stable as long as these key genes are retained. Such phenomena also agree well with that the power law networks are robust with respect to deletion of random nodes, but fragile with respect to deletion of hubs [40]. Moreover, by deleting or importing additional interactions at different percentages in the angiogenesis network, we found that the NIMS outputs were quite stable even when 50% of the edges were randomly removed or added (Figure 3c), indicating that NIMS is insensitive to both incompleteness and noise regarding the background network.

### Comparison with meet/min

To determine whether the synergy rank of agent pairs could be obtained from corresponding *agent genes* alone, regardless of network knowledge, we used the meet/min method, a similarity measurement between two gene sets that discards the network information [41], to rank the agent pairs. The meet/min method is believed to be simple but effective and non-biased [41]. Because the NIMS score and the meet/min coefficient (ranging from 0 to 1) will both reach their maximum when the gene set of one agent is merely the subset of that of the other agent, we only investigated agent combinations with valid scores from 0 to 0.9. In general, a relatively high correlation (SRCC=0.6251) between the meet/min coefficient and the NIMS synergy score was observed for all agent pairs. However, compared with the experimental results, the performance of the meet/min method was relatively poor in ranking synergistic pairs with Sinomenine (Table 2).

**Table 2:** Synergy ranks of five Sinomenine pairs resulted from NIMS, meet/min and cell experiment

Agent matched with Sinomenine	NIMS rank			The meet/min rank	Experimental rank
			
	*AS* × *TS*	*TS*	*AS*		
Matrine	4	5	10	20	1
Honokiol	8	6	32	19	2
Luteolin	10	21	12	21	3
Quercetin	14	14	26	29	4
Paeoniflorin	29	22	52	6	5

*AS*, Agent Score; *TS*, Topology Score.

### NIMS synergy and GO function

We measured Gene Ontology (GO) co-annotations to advance understanding of the underlying synergy mechanism for agent pairs predicted by NIMS. All three GO categories, Biological Processes, Cellular Components and Molecular Functions, were considered. As shown in Table 3, weak correlations were observed between the NIMS synergy scores and the GO similarity scores calculated from genes of each agent pairs. Results showed that agents with synergy may not target the same functional processes.

**Table 3:** Correlation of the NIMS synergy score with agent genes’ GO co-annotations

	Correlation of the NIMS score with GO similarity score		
	
Categories	Biological Processes	Cellular Components	Molecular Functions
SRCC	0.1649	0.0641	0.1571

p-value of SRCC	0.1963	0.617	0.2182

GO, Gene Ontology; SRCC, Spearman Rank Correlation Coefficient.

### Features of synergistic agent combinations on the angiogenesis network target

**Figure 4:**
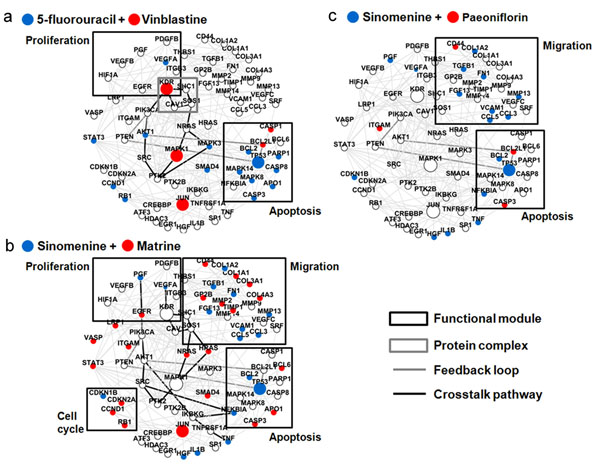
**Features of synergistic agent combinations on the angiogenesis network target. a**. 5-flourourcil and Vinblastine with known synergy. **b**. Sinomenine and Matrine with the high NIMS synergy score. **c**. Sinomenine and Paeoniflorin with the low NIMS synergy score. The nodes with red or blue colour denote responsive genes associated with two agents respectively.

Practically, we treat the angiogenesis network target as core subnetworks of angiogenesis network which contains the intersection of a set of shortest path subnetworks associated with individual or combinational drug actions. To learn the exact features on the angiogenesis network target derived from agent combinations with different NIMS scores, we mapped the responsive genes of 5-flourourcil, Vinblastine, Sinomenine, Matrine and Paeoniflorin to the network target and the detailed network features especially pathway crosstalks and feedback loops were analyzed. As shown in Figure 4, we found that the network target could capture different synergistic responses induced by three agent combinations with different NIMS synery scores. For example, 5-flourourcil and Vinblastine can affect KDR protein complex, the crosstalk between AKT1 and MAPK1 pathways, the PTEN feedback loop as well as two biological processes, endothelial cell proliferation and apoptosis, and four hub nodes (KDR, MAPK1, JUN and TP53). The network target affected by Sinomenine and Matrine contains the crosstalk with EGFR, KDR and TNFRSF1A pathways, the PTEN feedback loop, as well as, four biological processes closely associated with angiogenesis and two hub nodes (JUN and TP53). However, Sinomenine and Paeoniflorin with lower synergy score can only affect two biological processes and one hub node (TP53) (Figure 4).

### Characterizing the mechanisms of multicomponent synergy from a network target perspective

**Figure 5:**
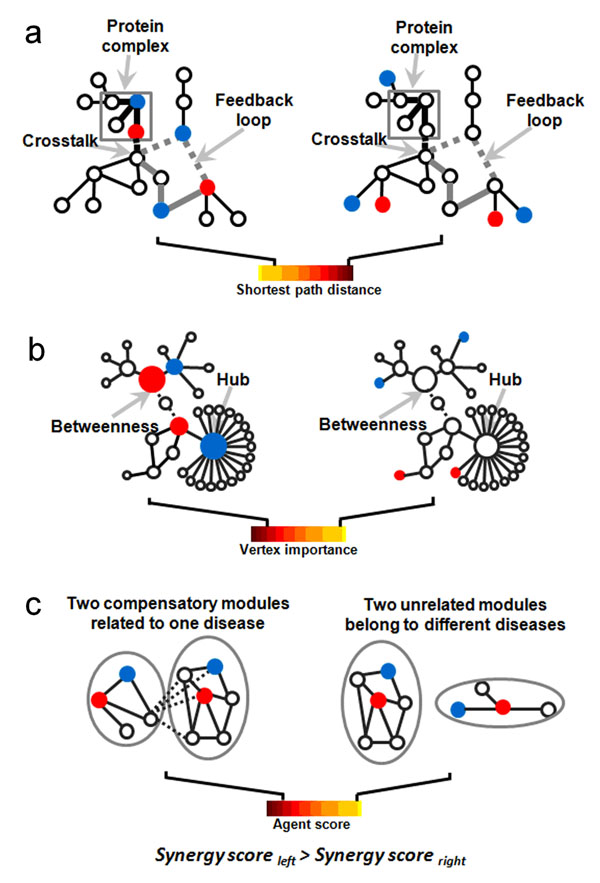
**A network target perspective for understanding the mechanisms of multicomponent synergy. a.** Two agents targeting a protein complex, the feedback loop or crosstalk in a signaling network (the left figure) may have the shortest path distance and obtain high NIMS synergy score compared to those do not (the right figure). **b**. Two agents targeting hub or betweenness nodes (the left figure) may produce higher synergism than the combinations targeting peripheral nodes (the right figure). **c**. Two agents targeting two compensatory modules related to one disease or the similar diseases (the left figure) may produce higher synergism than those targeting two unrelated modules from unrelated diseases (the right figure). Dashed lines represent direct or indirect connections in a network. Blue or red nodes denote the responsive genes of two agents respectively.

Despite the widespread occurrence of multicomponent therapeutics, the molecular mechanisms that underlie drug synergy remain unclear. Based on the above computational and experimental results of NIMS, we demonstrate that the network target can nicely interpret the multicomponent synergy by its latent network topology properties. We hence give a generalization of the network target concept and NIMS parameters to formalize our viewpoints on drug synergistic mechanisms. As shown in Figure 5, the shortest path distance (min(*d_i_*_,_*_j_*) in NIMS) can describe the protein complexes, crosstalks as well as feedback loops in the network formed by genes associated with two agents (Figure 5a), the hub and betweenness (*IP*(*v*) in NIMS) denotes the importance of genes or stimuli-influenced number of molecules two agents affected (Figure 5b), and functional modules means the biological processes two agents targeted (Figure 5c). It is important to note that these findings match well with the synergy phenomena present in complex biological systems. The available evidences showed that molecular synergisms can be emerged from different aspects, for example, protein complexes in cell-regulatory systems [42], crosstalk [43-47] and feedback control in the structures of signal pathways [48,49], stimuli-influenced number of molecules (e.g. number of activated enzymes, receptors, channels or transcription factors) [50,51] and gene expression profile [52] in signal transduction process. Thus, from the network target perspective, we can gain a comprehensive understanding of drug synergistic mechanisms on the basis of complex biological systems.

## Discussion

Recently, with the growing understanding of complex diseases, the focus of drug discovery has shifted from the well-accepted “one target, one drug” model designed toward a single target to a new “multi-target, multi-drug” model aimed at systemically modulating multiple targets [19,53]. In this work, we proposed the concept of “network target”, which treats the disease-specific biological network and its key elements as a therapeutic target, and established a NIMS approach to prioritize the multicomponent synergy. NIMS combines network topology and agent similarity, with regard to agent genes as well as phenotypes. To demonstrate the capability of NIMS, we applied this algorithm to the prioritization of synergistic anti-angiogenesis agent pairs from an empirical multicomponent therapeutic system, TCM. Our results show that NIMS, especially when used against the angiogenesis network, could not only successfully recover known synergistic drug pairs (Table 1), but also rank the anti-angiogenesis synergistic agents matched with a given agent, Sinomenine (Figure 2). Interestingly, two synergistic agent pairs predicted by NIMS in the case study, Sinomenine and Matrine, and Sinomenine and Honokiol, respectively, are main constituents of TCM herbal formulae such as *Qing-Luo-Yin*[*38*] and *Tou-Gu-Zhen-Feng* pill. These preliminary results demonstrate the potential of NIMS as a tool for screening synergistic combinations from current drugs as well as TCM herbs or herbal formulae.

NIMS uses the agent gene and phenotype information plus network topology features. We demonstrated that NIMS is robust to the collected *agent genes* if the key genes are reserved (Figure 3a and Figure 3b). Moreover, NIMS is also relatively robust to the background network, although available networks such as the PPI network are still incomplete and biased (Figure 3c) [54]. We consider the following aspects of NIMS may contribute to such robust performances. (1) The gene set information of agents not only reflects the knowledge of agent action similarity, but also determines the meet/min coefficient. We detected a potential correlation between the meet/min coefficient and the NIMS score. Thus the agent gene information itself ensures a relatively stable performance of NIMS against different types of networks. (2) The inherent agreement of topological features, a critical element in ranking synergistic agent pairs, is embedded in the angiogenesis, HPRD and KNC networks. On the contrary, poor performance is seen when the network topology is fundamentally altered, as in the MNC pathway network (Table 1). Note that the MNC pathway network is constructed in a different way (**See Methods for details**). (3) NIMS only makes use of a small fraction of the network around the network targets. Thus, it is relatively insensitive to changes of the whole background network but very sensitive to changes in key genes. This fact underlines the importance of the network target as a determining factor responsible for both disease mechanisms and agent actions in a network level.

We also evaluated the underlying synergy strengths produced by agent pairs from the perspective of GO functions. For 62 agents matched with Sinomenine, there is relatively lower correlation between NIMS synergy scores and GO co-annotations (Table 3). This finding is not surprising, because synergistic effects in multicomponent therapeutics could be achieved by genes that are involved in different biological processes related to a disease [1]. A disease or pathological condition is also characterized by the involvement of complex biological processes with hierarchical organization. Hence, synergistic agent pairs may not be restricted to act on the same biological functions.

Based on the above results, we further investigated the effects on the angiogenesis network target illustrated by three agent combinations with different NIMS synergy scores, namely 5-flourourcil and Vinblastine, Sinomenine and Matrine, and Sinomenine and Paeoniflorin (Figure 4), and characterized the multicomponent synergistic mechanisms from a network target perspective (Figure 5). Interestingly, we found that the network target coupling with NIMS parameters can capture the potential drug synergistic mechanisms from many aspects covering protein complexes, crosstalk and feedback loop of pathways, and stimuli-influenced molecular number [42-52], demonstrating the network target could serve as a new mode of drug target and the NIMS method is reasonable for identifying drug synergy. Such features also make NIMS compatible and upgradeable with other small-scale or large-scale network methods regarding drug action mechanisms we developed recently [9,55][22].

NIMS is a vital part in our NIDA (Network target-based Identification of Drug Action and drug synergy) system [56]. In previous studies, we demonstrate that this system can also be used to prioritize effects of candidate drugs / herbs on one or more biological processes related to given diseases [57]. To improve further the quality and performance of NIMS, there are three issues to be considered. First, the network target for a specific disease can be generated by disease-causal gene networks, disease-responsive gene networks or drug target networks. Due to the lack of understanding of complex diseases, here we only adopt the responsive gene network associated with a given disease or pathological process such as angiogenesis. It is believed that the more precise the network target is chosen, the more accurate predictions will be obtained, as suggested by the comparison results between the angiogenesis network and three global networks. We will also evaluate more useful parameters such as subgraph centrality and information centrality to calculate the node importance in both directed and undirected networks [58]. Additionally, the prediction obtained by NIMS may also be improved if we make use of more information such as the network Yin-Yang imbalance [25] or the side-effect information to refine the network target.

Second, though we only conducted the pure compounds to experimental studies, NIMS actually can be flexibly used to multiple ingredients in each herb as long as the related genes (*agent genes*) are available and reliable. To extend NIMS to more complicated conditions or more than two agents, we can treat mixed agents such as herb extracts and herbs as a group of compounds, and the predicted ranks of NIMS depend only on what *agent genes* are inputted and how accurate the *agent genes* are. For *agent genes*, the present work merely considered responsive genes associated with a limited number of TCM agents. Hopefully, NIMS can be updated when more precise information on drug targets is revealed for more agents by experiments or recent developed prediction tools such as drugCIPHER [55].

Third, as an initial effort for prioritizing synergistic agent combination in a computational framework, NIMS currently is a little bit simplified since it considers only part of the synergistic effects at the molecular level and currently does not make the distinction between the synergistic and antagonistic effects. The tissue-level synergism did not enter into our calculations. Further studies will be devoted to quantitative analysis of synergy, tissue-level synergy analysis, and pattern comparison between synergism and antagonism by integrating multilayer -omic data and spatio-temporal information. The identification of the cooperative behaviours and mechanisms of multiple agents as well as corresponding network targets will also be examined by both *in vitro* and *in vivo* experiments.

## Conclusions

In summary, our work demonstrates that the network target-based methods are of importance for estimating synergistic combinations and facilitating the combinational drug development. NIMS can serve as a first-step computational approach for the high-throughput identification of multicomponent synergy and the modernization of traditional Chinese medicine. It is also a promising way to elucidate the inter-relationship between complex diseases and drug interventions through the network target paradigm.

## Methods

### Data preparation

To obtain the empirical multicomponent candidates, 49 TCM herbs and 12 herb-derived compounds with potential anti-inflammatory, anti-angiogenic or anti-tumor activities were selected from the 2005 Edition of Chinese Pharmacopoeia, an official compendium of drugs, covering traditional Chinese herbs, herbal formulae and western medicines.Two chemicals 5-fluorouracil and Rapamycin were also included and resulted in a total of 63 agents. Five agent pairs among them were reported synergistic action and retrieved as benchmark data for NIMS outputs. By reading more than 2,000 references regarding agent actions from both PubMed and the China National Knowledge Infrastructure (http://www.cnki.net), available *agent genes* and *agent phenotypes* were manually collected. The number of genes for each agent ranged from 10 to 108. A total of 736 non-redundant *agent genes* were obtained. For calculating Agent Score (*AS*), we collected the *agent phenotype* similarity scores from the study of van Driel *et al*[*31*], in which the similarity score between two phenotypes is determined by the cosine of their feature vector angle, and the reliability of the score has also been tested [31].

### Angiogenesis network construction and three global networks

The angiogenesis gene network was constructed by the LMMA method we developed previously [17]. By using the keyword “Angiogenesis OR Neovascularization”, we retrieved 49,885 PubMed abstracts (until Feb 9, 2007), in which 2,707 genes were identified with Entrez gene ids and served as nodes of the angiogenesis network. Two genes were considered linked if they had any relationship in the PPI from HPRD (release 7) [59] or pathway interactions from KEGG [60]. We also employed three types of global networks, the PPI network and two types of global pathway networks merged from 201 KEGG human pathways, to evaluate the robustness of NIMS in terms of the background network. In KEGG, one node within a KEGG Orthology (KO) may denote a group of genes/proteins, and one gene may belong to different KOs. For example, K01090 contains 26 human genes, and the gene CDKN3 is categorized in both K01090 and K01104. Therefore, we built two distinct pathway networks: the Keep Node Content pathway network and the Merge Node Content pathway network. In the KNC network, the original node content was kept consistent, whereas in the MNC network, different KOs with one or more overlapping genes were merged into one node.

### NIMS robustness analysis

By changing the parameters and then calculating the correlation between the new and original NIMS scores, we checked whether NIMS could perform robustly. All three centrality measures (Betweenness, Closeness and PageRank) for *TS* and the role of *AS* were analyzed. Then, we conducted permutation tests and measured SRCC between the permutated and original *TS* or *AS* scores for the changes of collected *agent genes* as well as the background networks. In this step, *agent genes* were removed or added randomly from the angiogenesis network, changing 10% of the genes at a time. Each iteration of adding or removing genes was repeated 100 times. For angiogenesis network, we randomly deleted edges and imported additional edges respectively at different percentages, each repeated 20 times, and measured the synergy score.

### NIMS synergy and GO function analysis

To examine the association between biological functions and the NIMS predicited synergy, we used permutation tests and SRCC to evaluate whether the genes related to the synergistic agent pairs predicted by NIMS tended to have co-annotations in GO [61]. We used the Union-Intersection (*UI*) score to analyze the GO functional similarity for genes from each agent pair. The *UI* score was calculated by the GOstats package in Bioconductor [62], defined as , where *GOs*_*g*_1_ and *GOs*_*g*_2_ are the GO annotation term sets of *agent_1_ genes* and *agent_2_ genes*, respectively.

### Angiogenesis *in vitro* assay

We employed the commonly-used Endothelial Cell Proliferation assay to verify NIMS predicted synergistic effects on angiogenesis. Human Umbilical Vein Endothelial Cells were obtained from Cascade Biologics (Portland, USA), cultured in Medium 200 (Cascade Biologics), supplemented with low serum growth supplement including 2% fetal bovine serum and a well-documented angiogenic growth factor bFGF (5 ng/ml) stimulus. Sinomenine and the sampled partner agents were purchased from the National Institute for the Control of Pharmaceutical and Biological Products, Beijing, China. The concentration range of each agent was obtained from literature and the IC50 value (the half maximal inhibitory concentration) for each individual agent was measured. To compare the interacted agents under the same effect level, we determined the proportion of each agent pair by following the same ratio of the two agent’s IC50 values. For example, if the IC50 values of *agent_1_* and *agent_2_* are 10 and 100 respectively, we set the proportion of this agent pair as 1:10 in verification experiments. Each treatment was administrated after cell growth for 24 hours in a 96-well plate. Cell proliferation was estimated using a Cell Counting Kit (CCK-8, Dojindo, Japan) after 48 hours of treatment. Each experiment was repeated three times. By using the Bliss independence model [5], the synergistic strength was determined by calculating: *MIIR*=*max*(*IR_syn_*–*IR_add_*), where *IR_syn_* and *IR_add_* denote inhibition rates and the Bliss additive value of an agent pair at a certain dose/ratio.

## Competing interests

The authors declare that they have no competing interests.

## Authors' contributions

SL conceived and designed the experiments, analyzed the data and wrote manuscript. BZ participated in cell experiments, data analysis and writing manuscript. NZ participated in computational works.
